# Tumor P70S6K hyperactivation is inversely associated with tumor-infiltrating lymphocytes in triple-negative breast cancer

**DOI:** 10.1007/s12094-022-03006-3

**Published:** 2022-12-12

**Authors:** Rebeca Jimeno, Silvana Mouron, Roberto Salgado, Sherene Loi, Belén Pérez-Mies, Rodrigo Sánchez-Bayona, Luis Manso, Mario Martínez, Ana Garrido-García, Rosario Serrano-Pardo, Ramón Colomer, Miguel Quintela-Fandino

**Affiliations:** 1 Breast Cancer Clinical Research Unit, Clinical Research Program, CNIO, Madrid, Spain; 2 Peter MacCallum Cancer Centre, Melbourne, VIC Australia; 3 Department of Pathology, GZA-ZNA, Antwerp, Belgium; 4 Sir Peter MacCallum Cancer Department of Oncology, University of Melbourne, Melbourne, VIC Australia; 5 Department of Pathology, Hospital Universitario Ramón y Cajal, Instituto Ramón y Cajal de Investigación Sanitaria (IRyCIS), Madrid, Spain; 6 Faculty of Medicine, Universidad de Alcalá, Alcalá de Henares, Spain; 7 CIBERONC, Madrid, Spain; 8 Department of Medical Oncology, Hospital Universitario 12 de Octubre, Madrid, Spain; 9 Department of Pathology, Hospital Universitario 12 de Octubre, Madrid, Spain; 10 Department of Medical Oncology, Hospital Universitario La Princesa, Madrid, Spain; 11 Department of Pathology, Hospital Universitario La Princesa, Madrid, Spain; 12 Department of Medicine, Universidad Autónoma de Madrid, Madrid, Spain; 13 Medical Oncology, Hospital Universitario de Fuenlabrada, Madrid, Spain; 14 Endowed Chair of Personalised Precision Medicine, Department of Medicine, Universidad Autónoma de Madrid, Madrid, Spain

**Keywords:** TNBC, P70S6K, TIL, T cells, B cells

## Abstract

**Purpose:**

Triple-negative breast cancer (TNBC) is characterized by large heterogeneity and relative lack of available targeted therapies. To find therapeutic strategies for distinct patients with TNBC, several approaches have been used for TNBC clustering, including recently immune and phosphoproteomic patterns. Based on 70-kDa ribosomal protein S6 kinase (P70S6K)-TNBC clustering, the current study explores the immune profiling in TNBC tumors.

**Methods:**

Stromal tumor-infiltrating lymphocytes (sTILs) were evaluated in human TNBC tumor samples. Furthermore, immunohistochemistry staining for CD8, CD4, Foxp3, and CD20 was performed in tissue microarrays (TMA) sections.

**Results:**

Histological analysis showed decreased sTILs, CD20^+^ cells, and CD8^+^/CD4^+^ ratio in high phosphorylated P70S6K (p-P70S6K) tumors. Moreover, p-P70S6K score was directly correlated with CD4^+^ and Foxp3^+^ T cells, while it was inversely correlated with CD8^+^/CD4^+^ and CD8^+^/Foxp3^+^ ratios.

**Conclusion:**

sTIL infiltration and lymphocyte profiling vary in the context of hyperactivation of P70S6K in TNBC tumors.

## Introduction

Triple-negative breast cancer (TNBC) is an aggressive breast cancer (BC) subtype frequently associated to rapid progression and high rate of early recurrences and metastasis in brain, liver, and lung [1–5]. Due to its poor differentiation and molecular heterogeneity, TNBC is challenging to treat and responses to treatment are frequently short-lasting.

Several approaches based on gene expression patterns, transcriptomic profiling and other strategies have been implemented for TNBC clustering. Lately, immunogenic profile has been used to classify TNBC tumors, which have shown the highest immune infiltration among BC subtypes [6, 7]. A recent study has confirmed the prognostic role of tumor-infiltrating lymphocytes (TILs) in TNBC [8]. However, some challenges remain in establishing the analytical and clinical validity of TILs in BC.

Trying to identify new targets that allow stratification of TNBC, we have previously described a functional clustering based on kinase activity profiling [9]. One of the kinases described was the 70-kDa ribosomal protein S6 kinase (P70S6K) whose hyperactivation was associated with adverse outcome [9]. P70S6K is a serine/threonine kinase triggered downstream of the PI3K/AKT/mTOR pathway, which is often activated in patients with TNBC [10–13]. P70S6K is overexpressed in cell lines and TNBC clinical samples and it has been proposed as a mediator of cancer cell migration, invasion, and metastasis [14–18].

Most of TNBC clustering approaches are based on a single strategy; however, given the heterogeneity of this BC subtype, combined information from different clustering patterns could provide a more rational stratification system. The present study relies on P70S6K profiling to characterize the immune signature of patients with TNBC.

## Material and methods

### Tumor samples

Female patients with a diagnosis of primary, non-metastatic BC, with expression of estrogen receptor (ER) and/or progesterone receptor (PR) < 1% and lack of human epidermal growth factor receptor 2 (HER2) amplification diagnosed at *Hospital 12 de Octubre*, *Hospital de Fuenlabrada* and *Hospital* *La Princesa* were eligible for this study. HER2 amplification was defined as a HER2/CEP17 ratio > 2 in FISH or a value of +++/+++ in an HERCEPTEST. Samples were obtained before the administration of any treatment.

### Immunohistochemistry analysis

Tumor samples were included in fresh 10% neutral buffered formalin immediately after sample collection. TMA were mounted with two 1.5 mm cores per sample. Deparaffinization and antigen retrieval were performed in an automated immunostaining platform (Discovery XT-ULTRA, Leica) using validated reagents (Ventana, Roche). The following antibodies were used: phospho-p70S6K (Thr389) (1A5); CD8 (C8/144B); CD4 (4B12); Foxp3 (236A/E7); CD20 (L26). Whole slides were acquired with a slide scanner (AxioScan Z1, Zeiss). Digital images were analyzed with image analysis system Zen Blue Software (V 3.1, Zeiss). In case of p-P70S6K staining, an H-score was generated as described previously [9]. For normalized data, a *Z*-score of p-P70S6K was calculated for each hospital and staining run. For immune infiltrate characterization, percentage of each lymphocyte marker was calculated as total positive area normalized to total area. Regarding TILs quantification, percentage of stromal TILs (sTILs) was evaluated from hematoxylin and eosin (H&E) stained sections of tumor samples according to the International Immuno-Oncology Working Group Guidelines [19–22].

### Statistics

Statistical analyses were performed using Prism (Prism 8.3.0, GraphPad software). Differences between groups were determined using unpaired two-tailed Student’s *t* test. Correlation analyses were performed using Spearman’s test.

## Results

To characterize the tumor lymphocyte infiltrate in the context of hyperactivation of P70S6K, TILs quantification and profiling were performed in primary samples of 341 patients with TNBC. Demographic information and characteristics of patients with TNBC are presented in Table 1.

**Table 1:** Clinical and demographical characteristic of patients with TNBC

Characteristic	Number (%)
Age (median, range)	52.96
	(24.73–89.50)
Subtype	
ER and/or PR positive, HER2 non-amplified^a^	0 (0%)
HER2-amplified, any ER/PR	0 (0%)
ER, PR, and HER2 negative	341 (100%)
Tumor size	
T1	48 (14.1%)
T2	150 (44.0%)
T3	38 (11.1%)
T4	22 (6.5%)
N/A^b^	83 (24.3%)
Nodal stage	
N0	140 (41.1%)
N1	56 (16.4%)
N2	37 (10.9%)
N3	21 (6.2%)
N/A	87(25.5%)
Grade	
G1	5 (1.5%)
G2	57 (16.7%)
G3	206 (60.4%)
N/A	73 (21.4%)
Chemotherapy	
None	2 (0.6%)
Neoadjuvant	116 (34.0%)
Adjuvant	216 (63.3%)
Neoadjuvant + adjuvant	7 (2.1%)
Chemotherapy regimen	
None	2 (0.6%)
CMF^c^	34 (10.0%)
Included anthracyclines	70 (20.5%)
Included anthracyclines and taxanes	119 (34.9%)
Included carboplatin	33 (9.7%)
N/A	83 (24.3%)

^a^Estrogen receptor (ER), progesterone receptor (PR), human epidermal growth factor receptor 2 (HER2)

^b^N/A: non-available

^c^CMF: cyclophosphamide plus methotrexate plus 5-fluorouracil

**Fig. 1:**
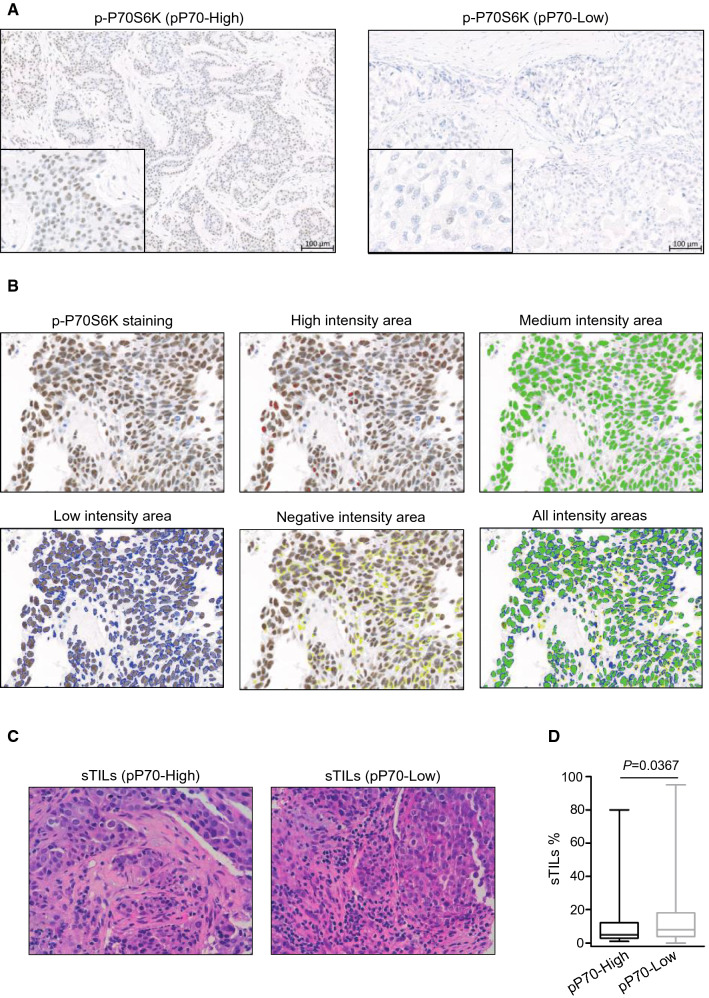
Microscopic evaluation of p-P70S6K and tumor-infiltrating lymphocytes in patients with TNBC. **A** Representative immunohistochemistry images of pP70-High (left) and pP70-Low (right) from TMA cores of TNBC tumor samples. Bar indicates 100 µm. **B** Representative images of p-P70S6K staining analysis. H-Score was generated as previously published [9] considering areas of high staining (red, Area_Color 1), medium staining (green, Area_Color 2), and low staining (blue, Area_Color 3). Percentage of each staining per sample was normalized to total area, including the negative-staining area (yellow), providing a computerized H-score calculated by formula: ((% of Area_Color1 × 3) + (% of Area_Color2 × 2) + (% of Area_Color3 × 1))/100. Images were acquired with a slide scanner (AxioScan Z1, Zeiss) and photomicrographs were obtained using the image analysis system Zen Blue Software (V3.1). **C** Representative images of H&E staining of pP70-High (left) and pP70-Low (right) TNBC tumor cases. Magnification ×20. Images were acquired with digital microscope Leica DMD 108 from LeicaMicrosystems. **D** Percentage of sTILs was determined by expert pathologists following recommendation guidelines. Box and Whiskers plots are shown for sTILs percentage in pP70-High (dark) or pP70-Low (grey) specimens. Unpaired two-tailed *t* test with Welch’s correction was performed and statistically significant *P* value is shown. *n* = 82 pP70-High; *n* = 244 pP70-Low

Phosphorylation levels of P70S6K were determined by immunohistochemistry staining as previously published [9]. H-Score of p-P70S6K expression was calculated using an image analysis software and samples were classified as high p-P70S6K (herein “pP70-High”; including samples in the upper quartile) or low p-P70S6K (herein “pP70-Low”; the remaining patients). Photomicrographs of representative stained tissue samples and analysis are shown (Fig. 1A, B). Tumor immune infiltration was studied by evaluation of sTILs (Fig. 1C). Percentage range of sTILs (0%, 1–9%, 10–20%, > 20%) and p-P70S6K status distributions according to clinical characteristic of patients are shown in Table 2. Changes in tumor infiltration were found between both types of patients as sTILs percentage showed a significant decrease in those patients exhibiting hyperactivation of P70S6K (12.40 ± 1.965 pP70-High; 17.70 ± 1.574 pP70-Low) (Fig. 1D).

**Table 2:** sTILs percentage, p-P70S6K status, and clinical characteristic of patients with TNBC

Clinical characteristic	TILs range	Number (%)	p-P70S6K status	Number (%)
Tumor size	T1-T2		T1–T2	
	0%	1 (0.5%)	pP70-High	52 (26.3%)
	1–9%	94 (47.5%)	pP70-Low	146 (73.7%)
	10–20%	42 (21.2%)		
	> 20%	51 (25.8%)		
	N/A	10 (5.0%)		
	T3–T4		T3–T4	
	0%	0 (0.0%)	pP70-High	10 (16.7%)
	1–9%	34 (56.7%)	pP70-Low	50 (83.3%)
	10–20%	18 (30.0%)		
	> 20%	3 (5.0%)		
	N/A	5 (8.3%)		
Nodal stage	N0–N1		N0–N1	
	0%	1 (0.5%)	pP70-High	54 (27.6%)
	1–9%	99 (50.5%)	pP70-Low	142 (72.4%)
	10–20%	47 (24.0%)		
	> 20%	39 (19.9%)		
	N/A	10 (5.1%)		
	N2–N3		N2–N3	
	0%	0 (0.0%)	pP70-High	7 (12.1%)
	1–9%	27 (46.6%)	pP70-Low	51 (87.9%)
	10–20%	14 (24.1%)		
	> 20%	13 (22.4%)		
	N/A	4 (6.9%)		
Grade	G1–G2		G1–G2	
	0%	1 (1.6%)	pP70-High	13 (21.0%)
	1–9%	36 (58.1%)	pP70-Low	49 (79.0%)
	10–20%	13 (21.0%)		
	> 20%	9 (14.5%)		
	N/A	3 (4.8%)		
	G3		G3	
	0%	2 (1.0%)	pP70-High	53 (25.7%)
	1–9%	105 (51.0%)	pP70-Low	153 (74.3%)
	10–20%	47 (22.8%)		
	> 20%	40 (19.4%)		
	N/A	12 (5.8%)		

**Fig. 2:**
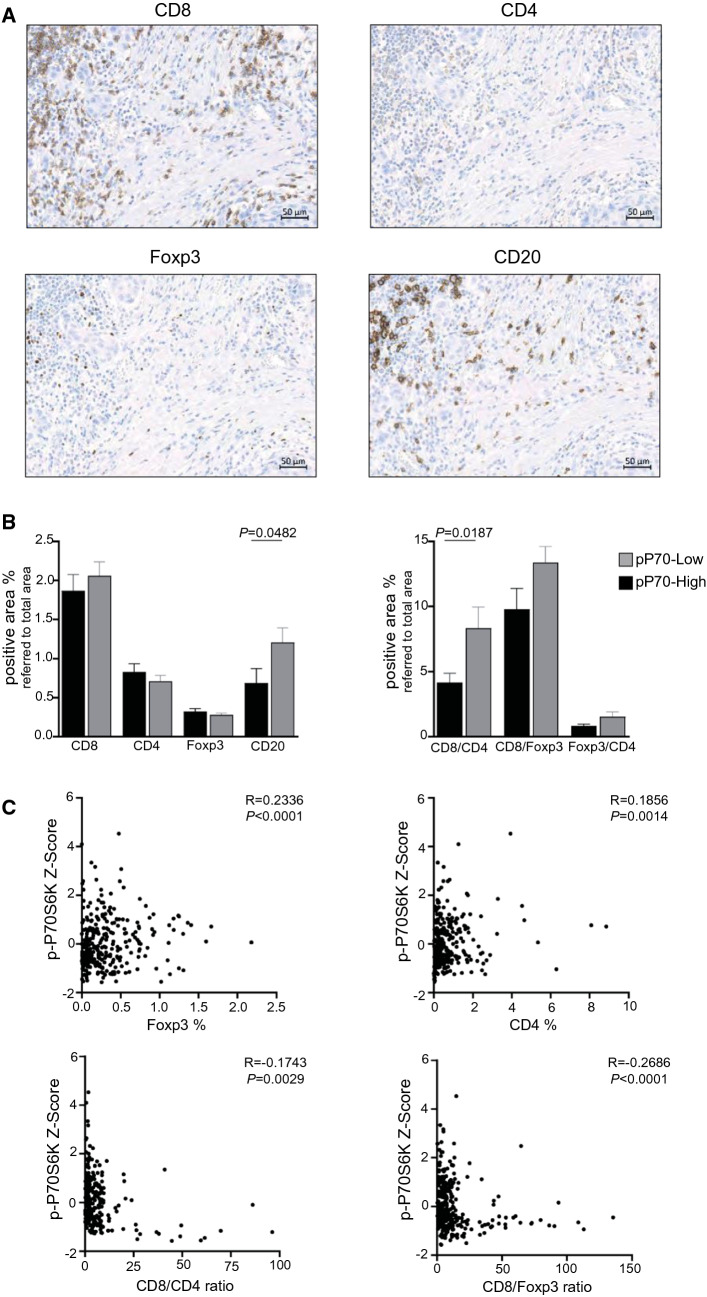
CD8^+^ and CD4^+^ T cells, Tregs and B cells quantification in pP70-High and pP70-Low TNBC. **A** Representative immunohistochemistry images of an area showing CD8, CD4, Foxp3, and CD20 staining. Bar indicates 50 µm. Images were acquired with a slide scanner (AxioScan Z1, Zeiss) and photomicrographs were obtained using the image analysis system Zen Blue Software (V3.1). **B** Mean values of percentage of CD8^+^, CD4^+^, Foxp3^+^, and CD20^+^ (left) and mean values of CD8^+^/CD4^+^, CD8^+^/Foxp3^+^, and Foxp3^+^/CD4^+^ ratios (right) in pP70-High (dark) or pP70-Low (grey) specimens. *n* = 74–81 pP70-High; *n* = 213–241 pP70-Low. Data were calculated using an automated scanning microscope (AxioScan Z1, Zeiss) and computerized image analysis system (Zen Blue Software V3.1). Percentage of positive area (intraepithelial and stromal) from each lymphocyte population is referred to total core area. Plots show mean ± SEM. Unpaired two-tailed *t* test with/without Welch’s correction was performed and statistically significant *P* values are shown. **C** Spearman’s correlation between p-P70S6K and each lymphocyte marker and ratio was determined. Individual correlation plots are shown for statistically significant results. Spearman’s coefficient of the correlation between the two markers is shown (*R* and *P* values). *n* = 286–321

Given the functional heterogeneity of tumor lymphocytic infiltrate, individual characterization of lymphocyte populations was next performed to add specificity to the value of sTILs estimation. Single immunohistochemical staining of CD8^+^, CD4^+^, Foxp3^+^, and CD20^+^ cells was used for analysis of cytotoxic T cells, total helper T cells, regulatory T cells (Tregs), and B cells, respectively (Fig. 2A). When comparing pP70-High and pP70-Low cases, a significant decrease in CD20^+^ cells was seen in high phosphorylated samples (Fig. 2B). However, no significant differences were found in T lymphocyte populations between both types of samples (Fig. 2B).

As the equilibrium between different T cell subsets is involved in the final outcome of the tumor immune response, CD8^+^/CD4^+^, CD8^+^/Foxp3^+^, and Foxp3^+^/CD4^+^ ratios were calculated. Significant decrease of CD8^+^/CD4^+^ ratio was seen in pP70-High samples (Fig. 2B).

Additionally, monotonic relationships between each population and p-P70S6K score were analyzed. While continuous Foxp3 and CD4 expression levels were positively correlated with p-P70S6K status, negative correlations were found for CD8^+^/CD4^+^ and CD8^+^/Foxp3^+^ ratios (Fig. 2C).

## Discussion

The lack of classical histologic biomarkers and the huge heterogeneity of TNBC make the profiling of this BC subtype necessary for defining different treatment strategies. Several approaches have been investigated for TNBC stratification, including lately those based on the prognostic role of TILs to describe distinct response subtypes of TNBC following adjuvant chemotherapy [8]. Recently, we have also described the prognostic impact of hyperactivation of P70S6K and other kinases on disease outcome and risk of relapse of patients with TNBC [9]. Based on our previous study, we decided to investigate the relationship between tumor P70S6K hyperactivation and immune infiltration in TNBC. First, we broadly examined the immune infiltrate by determining sTILs in tumors expressing high or low phosphorylation levels of P70S6K. Results indicate a decreased sTILs percentage in those tumors overexpressing p-P70S6K. Given the association of TILs with good prognosis in TNBC [8], it is predictable that tumor microenvironment (TME) is less infiltrated in P70S6K hyperphosphorylated tumors which have previously shown worse prognosis [9]. Relationships between P70S6K expression in BC and TILs have not yet been clearly defined. A previous study in HER2-positive BC has shown that tumors with high levels of pS6 expression are associated with high percentage of TILs; however, the specific role of P70S6K was not determined [23].

Characterization of tumors as rich- and poor-TILs may not completely replicate distinct immune responses that can be initiated in the TME. Therefore, we further characterized immune subpopulations in our set of TNBC samples. We found significant decrease in CD20^+^ cells in pP70-High tumors, whereas not significant differences were found in T lymphocytes between both types of samples. However, a positive correlation was found for CD4 and Foxp3 expression and p-P70S6K score.

As different subsets of T cells may act in opposite ways against tumor progression, we also determined balances between them in pP70-High and pP70-Low samples. Analysis of ratios among different populations of T cells has previously demonstrated a great utility in TNBC. Several studies have reported association of CD8/Foxp3, CD8/CD4, and CD4/Foxp3 with improved survival of patients and response to different treatments [24–27]. However, while increased CD4/CD8 ratio was related with adverse prognosis in triple-negative invasive ductal carcinoma [28], another study has described an association between CD4/CD8 ratio and distant relapse-free survival and overall survival in TNBC [29]. In our cohort of patients with TNBC, a decreased CD8^+^/CD4^+^ ratio was seen in pP70-High tumors. Moreover, negative correlations for p-P70S6K score and CD8^+^/CD4^+^ and CD8^+^/Foxp3^+^ ratios were found. Although correlations do not indicate causation, our findings suggest that P70S6K activity may negatively influence pro-inflammatory CD8 immune responses while favoring regulatory T cell responses. Similarly, a previous study has suggested that deregulation of the PI3K pathway could contribute to immune escape by mitigating pro-inflammatory responses and increasing the percentage of Foxp3^+^ lymphocytes [30]. More functional studies are needed for a better understanding of precise mechanisms regulating T cell function developed by P70S6K in TNBC. A limitation of our study is that we did not illustrate the mechanisms by which P70S6K modulates the lymphoid landscape in TNBC tumors. Increasing the difficulty, the huge complexity of lymphocytes at both levels, cell phenotype and activation status, constitutes a challenge when studying T cell responses. Our analyses in TNBC patient-derived samples do not inform about specific lymphocyte phenotypes (i.e., different subsets of T helper cells) or their activation status and functionality. Further characterization of phenotypic or activation markers of lymphocytes should be considered when defining lymphocyte infiltrates in TNBC.

In brief, our study describes the lymphocyte profiling in the context of P70S6K hyperactivation in TNBC, providing valuable data to be considered for improved TNBC clustering. While during the last years, phosphoproteomic approaches are gaining attention in BC, how this strategy can be combined with new and promising immune-profiling approaches has to be further explored.

## Data Availability

The authors confirm that the data supporting the findings of this study are available within the article.
